# SHARE – workpackage 5: evidence based recommendations for diagnosis and treatment of juvenile dermatomyositis

**DOI:** 10.1186/1546-0096-12-S1-P89

**Published:** 2014-09-17

**Authors:** Felicitas Bellutti Enders, Brigitte Bader-Meunier, Eileen Baildam, Tamas Constantin, Pavla Dolezalova, Brian Feldman, Pekka Lahdenne, Bo Magnusson, Kiran Nistala, Clarissa Pilkington, Angelo Ravelli, Ricardo Russo, Marco van Brussel, JanJaap van der Net, Lucy Wedderburn, Bas Vastert, Nico Wulffraat, Liza McCann, Annet van Royen-Kerkhof

**Affiliations:** 1 Department of Pediatric Immunology, University Medical Centre Utrecht, Utrecht, Netherlands; 2 Department for Immunology, Hematology and Pediatric Rheumatology, Necker Hospital, Paris, France; 3 Pediatric Rheumatology, Alder Hey Children’s NHS Foundation Trust, Liverpool, UK; 4 Pediatric Rheumatology, Semmelweiss Hospital, Budapest, Hungary; 5 Department of Paediatrics and Adolescent Medicine, Charles University, Prague, Czech Republic; 6 Department of Rheumatology, , The Hospital for Sick Children, Toronto, Canada; 7 Pediatric Rheumatology, Children’s Hospital, Helsinki University Central Hospital, Helsinki, Finland; 8 Pediatric Rheumatology, Astrid Lindgren Children's Hospital, Karolinska University Hospital, Stockholm, Sweden; 9 Centre for Rheumatology, University College London, London, UK; 10 Pediatria II, Reumatologia, Istituto Giannina Gaslini, Genova, Italy; 11 Service of Immunology and Rheumatology, Hospital de Pediatría Garrahan, Buenos Aires, Argentina; 12 Child Development and Exercise Center, University Medical Center Utrecht, Utrecht, Netherlands

## Introduction

Juvenile Dermatomyositis is a rare *Pediatric Rheumatic Disease*, associated with significant morbidity. Evidence-based guidelines are sparse and management is mostly based on physician’s experience. Consequently, treatment regimens differ throughout Europe. In 2012, a European initiative called SHARE (**S**ingle **H**ub and **A**ccess point for pediatric **R**heumatology in **E**urope) was launched to optimize and disseminate diagnostic and management regimens in Europe for children and young adults with rheumatic diseases.

## Objectives

One of the aims of SHARE was to provide evidence-based recommendations for diagnosis and treatment of JDM.

## Methods

Evidence based recommendations were developed using the European League Against Rheumatism (EULAR) standard operating procedure ^(1)^. An expert committee was instituted, consisting of pediatric rheumatologists and experts in pediatric exercise physiology and physical therapy. The expert committee defined search terms for the systematic literature review. Two independent experts scored articles for validity and level of evidence. Recommendations derived from the literature were evaluated by an online survey. Those with less than 80% agreement during the online survey were reformulated. Subsequently, all recommendations were discussed at a consensus meeting using nominal group technique [2]. Recommendations were accepted if more than 80% agreement was reached.

## Results

The literature search yielded 3280 articles, of which 108 (63 for diagnosis and 44 for treatment) were considered relevant and therefore scored for validity and level of evidence. 77 articles (55 for diagnosis and 22 for treatment) were scored valid and used in the formulation of the recommendations. 21 recommendations for diagnosis and 7 for treatment were suggested in the online survey. During the consensus meeting, recommendations were discussed and re-formulated where applicable. It was agreed that all children with suspected inflammatory myopathies should be referred to a specialist centre, with immediate referral for defined high-risk patients. In addition to this, 20 evidence-based recommendations for diagnosis and 9 for treatment were accepted with more than 80% agreement. Topics covered for diagnosis, included assessment of skin involvement, muscle involvement including muscle biopsy, MRI and muscle testing, lung involvement, auto-antibodies, and biomarkers. Treatment recommendations included initial treatment of newly diagnosed patients and therapy for severe disease.

## Conclusion

The SHARE initiative provides recommendations for diagnosis and treatment for JDM and thereby facilitates improvement and uniformity of care throughout Europe.

## Disclosure of interest

None declared.

